# A Hybrid Fault Diagnosis Approach for Rotating Machinery with the Fusion of Entropy-Based Feature Extraction and SVM Optimized by a Chaos Quantum Sine Cosine Algorithm

**DOI:** 10.3390/e20090626

**Published:** 2018-08-21

**Authors:** Wenlong Fu, Jiawen Tan, Chaoshun Li, Zubing Zou, Qiankun Li, Tie Chen

**Affiliations:** 1 College of Electrical Engineering & New Energy, China Three Gorges University, Yichang 443002, China; 2 Hubei Provincial Key Laboratory for Operation and Control and Cascaded Hydropower Station, China Three Gorges University, Yichang 443002, China; 3 School of Hydropower and Information Engineering, Huazhong University of Science and Technology, Wuhan 430074, China; 4 China Three Gorges Corporation, Chengdu 610041, China

**Keywords:** fault diagnosis, variational mode decomposition, permutation entropy, Duffing system, chaos quantum sine cosine algorithm

## Abstract

As crucial equipment during industrial manufacture, the health status of rotating machinery affects the production efficiency and device safety. Hence, it is of great significance to diagnose rotating machinery faults, which can contribute to guarantee the running stability and plan for maintenance, thus promoting production efficiency and economic benefits. For this purpose, a hybrid fault diagnosis model with entropy-based feature extraction and SVM optimized by a chaos quantum sine cosine algorithm (CQSCA) is developed in this research. Firstly, the state-of-the-art variational mode decomposition (VMD) is utilized to decompose the vibration signals into sets of components, during which process the preset parameter *K* is confirmed with the central frequency observation method. Subsequently, the permutation entropy values of all components are computed to constitute the feature vectors corresponding to different kind of signals. Later, the newly developed sine cosine algorithm (SCA) is employed and improved with chaotic initialization by a Duffing system and quantum technique to optimize the support vector machine (SVM) model, with which the fault pattern is recognized. Additionally, the availability of the optimized SVM with CQSCA was revealed in pattern recognition experiments. Finally, the proposed hybrid fault diagnosis approach was employed for engineering applications as well as contrastive analysis. The comparative results show that the proposed method achieved the best training accuracy 99.5% and best testing accuracy 97.89%. Furthermore, it can be concluded from the boxplots of different diagnosis methods that the stability and precision of the proposed method is superior to those of others.

## 1. Introduction

Rotating machinery plays a significant role in modern industrial fields, and its health status greatly influences the production efficiency and product quality. Besides, once an unexpected or sudden fault occurs, it could result in large economic losses. Hence, it is of great practical significance to diagnose rotating machine faults [1]. During the running process of various kinds of rotating machines, rolling element bearings are the most widely used parts. However, owing to their structural properties and operating environment, rolling bearing damage is inevitable and will affect the mechanical properties of the equipment to some extent [2]. Therefore, some diagnostic measures need to be carried out for rolling element bearings, thus promoting the stable and efficient operation of rotating machines [3,4]. Generally, when a fault occurs in a rolling element, it is accompanied by a certain amount of vibration and sound. Thus, potential faults can be well detected with appropriate techniques for processing the collected vibration or acoustic signals [5,6]. Due to the fact that vibration signals carry rich information about potential faults, vibration analysis has been widely applied to diagnose the faults of rolling element bearings, which always includes two procedures: one is the feature extraction of vibration signals with signal processing techniques, and the other is the fault pattern recognition for the extracted features [7,8].

After collecting the vibration signals, the extraction of representative features is the main mission. However, the signals are always non-stationary and non-linear, which makes it difficult to extract the pivotal features effectively. To solve this problem, many time-frequency signal processing methods have been proposed in previous studies, such as wavelet transform (WT, [9]), empirical mode decomposition (EMD, [10]), ensemble empirical mode decomposition (EEMD, [11]) and variational mode decomposition (VMD, [12]). Among the above methods, WT [9] is an adaptive signal analysis method proposed based on the localization idea of the Fourier transform, possessing strong recognition ability for transient signals, and has been widely used to analyze non-stationary signals [13,14]. However, once the wavelet basis is designated, the generalization will be poor. EMD, originally proposed by Huang [10], decomposes a given signal into components with different scales through loop iteration, and possesses better adaptability since the decomposition only depends on the local characteristics of the signal. Though EMD has attracted great attention due to its ability to deal with non-stationary signals [15,16], its performance is severely affected by mode mixing and end effects. To solve these problems, an improved version-EEMD was put forward with noise assistance [11] and has become a focus in the field of signal processing [17,18]. Unlike EMD/EEMD that lack a mathematical theory foundation, the newly proposed VMD [12] is a quasi-orthogonal signal processing method which decomposes the given signal by solving a constrained variational problem. Besides, the effectiveness and superiority of VMD have been verified in previous studies [19,20].

With the non-stationarity of the vibration signals weakened by the aforementioned methods, it is necessary to extract fault features from the components. As it is known that information entropy is an effective indicator for measuring the uncertainty degree of signals, combining entropy theory with signal processing methods is expected to represent the fault characteristics well. For this reason, different entropy methods, including energy entropy [21], sample entropy [22,23], approximate entropy [24,25], permutation entropy [26,27] and so on, have been utilized to solve feature extraction and fault diagnosis problems. For example, Xiao et al. [21] extracted the energy entropy of sub-band signals decomposed from the stator current of doubly-fed wind turbine with dual-tree complex wavelet transform. Zhang et al. [23] calculated the sample entropy of sub-bands of rolling bearings decomposed by lifting wavelet packet transform. An et al. [25] calculated the approximate entropy of the selected components of vibration signals from a wind turbine rolling bearing decomposed by adaptive local iterative filtering. Shi et al. [27] combined the improved local mean decomposition with permutation entropy to extract features. Among the above entropy methods, permutation entropy proposed by Bandt et al. [28] is a time series method, which can detect the dynamic catastrophic behavior. Due to the simple and fast calculation as well as strong anti-noise ability, permutation entropy was introduced to measure the status characterization of rotary machines [29]. Subsequently, it has been widely applied to extract features for fault diagnosis and shown outstanding performance [26,27].

During the fault pattern recognition stage, many machine learning methods have been proposed in the previous literatures, including *k*-nearest neighbor [30], Bayesian decision [31], artificial neural network (ANN, [32,33]), support vector machine (SVM, [34]) and so on. Among these recognition techniques, *k*-nearest neighbor is simple in theory and susceptible to sample distribution. Bayesian decision can acquire well performance with the consideration of priori probabilities. ANN has strong recognition ability when the number of samples is large. However, all three methods mentioned above are based on empirical risk minimization, i.e., abundant samples are needed to achieve high accuracy. In contrast, SVM, proposed by Vapnik [35] based on structural risk minimization, has certain advantages in dealing with small samples and linearly inseparable problems. However, the pattern recognition performance of SVM is influenced by the parameters. To solve the problem, different optimization methods, such as particle swarm optimization [36], antlion algorithm [37], fruit fly algorithm [38] and ant colony algorithm [39] were proposed and employed to choose the best parameters for SVM.

Sine cosine algorithm (SCA) is a newly developed optimization algorithm proposed by Mirjalili [40] that has shown good performance in many studies [41,42]. To achieve accurate fault diagnosis for rotating machinery, a hybrid fault diagnosis model with entropy-based feature extraction and SVM optimized by chaos quantum sine cosine algorithm (CQSCA) is developed in this research. Firstly, the adaptive VMD is employed to decompose the vibration signals into a set of components, during which stage the preset parameter *K* of VMD is ascertained with central frequency observation method. Then, the permutation entropy values of all the sub-signals are calculated, thus to construct the feature vector of the given fault sample. Subsequently, an improved SVM model with full fusion of chaotic initialization, quantum technique and SCA for parameter selection, whose effectiveness has been proved in pattern recognition experiments, is presented to classify different fault types. Finally, the superiority of the proposed method was confirmed through engineering applications as well as comparative analysis. 

The remainder of this paper is organized as follows: Section 2 presents the base theory of VMD and permutation entropy. Section 3 introduces the improved pattern recognition method based on SVM optimized with chaos quantum sine cosine algorithm and validates the effectiveness with pattern recognition experiment. Section 4 delineates the procedures of the proposed hybrid fault diagnosis model with entropy-based feature extraction and SVM optimized by CQSCA. Section 5 illustrates the superiority of the proposed method with engineering application and comparative analysis. The conclusions are summarized in Section 6.

## 2. Entropy-Based Feature Extraction with VMD 

### 2.1. Variational Mode Decomposition

VMD [12] is a newly developed time-frequency signal processing technique, which is adaptive and quasi-orthogonal. With VMD, a given signal can be decomposed into a set of intrinsic mode functions (IMF) which are all band-limited. The decomposition process can be realized by solving a constrained variational problem formulated as follows [12]:

(1) $$\begin{array}{l} \underset{m_{k},w_{k}}{\min\;}\{\sum_{k}{\left\|\partial_{t}\left[\left(\delta (t)+\frac{j}{\pi t}\right)*m_{k}(t)\right]e^{-jw_{k}t}\right\|}_{2}^{2}\}\\ s.t.\;\;\;\sum_{k=1}^{K}m_{k}=f,\;\;\;\;\;\;k=1,2,\ldots ,K \end{array}$$

where *K* is the total number of IMFs, *m_k_* is the time domain signal of the *k*-th IMF, and *w_k_* is the center pulsation of the *k*-th IMF.

To obtain the solution of Equation (1), a quadratic penalty term and a Lagrangian multiplier are introduced, and the augmented problem is provided as follows:

(2) $$\begin{array}{l} L(m_{k},w_{k},\beta )=\alpha \sum_{k}{\left\|\partial_{t}\left[\left(\delta (t)+\frac{j}{\pi t\;}\right)*m_{k}(t)\right]e^{-jw_{k}t}\right\|}_{2}^{2}+\;\\ \;\;\;\;\;\;\;\;\;\;\;\;\;\;\;\;{\left\|f(t)-\sum_{k}m_{k}(t)\right\|}_{2}^{2}+\langle \beta (t),f(t)-\sum_{k}m_{k}(t)\rangle \end{array}$$

where *α* and *β*(*t*) are respectively the balancing parameter and Lagrange multiplier.

Then the alternate direction method of multipliers (ADMM, [43]) is applied to deduce the solution of Equation (2) by optimizing *m_k_*, *w_k_* and *β* alternately, which is based on the ideas of Lagrange theory and dual decomposition. The optimization problems of *m_k_* and *w_k_* are respectively presented as Equations (3) and (4):

(3) $$m_{k}^{n+1\;}=\min\left\{\alpha {\left\|\partial_{t}\left[\left(\delta (t)+\frac{j}{\pi t}\right)*m_{k}(t)\right]e^{-jw_{k}t}\right\|}_{2}^{2}+{\left\|f(t)-\sum_{i}m_{i}(t)+\frac{\beta (t)}{2}\right\|}_{2}^{2}\right\}$$

(4) $$w_{k}^{n+1\;}=\min\left\{{\left\|\partial_{t}\left[\left(\delta (t)+\frac{j}{\pi t}\right)*m_{k}(t)\right]e^{-jw_{k}t}\right\|}_{2}^{2}\right\}$$

The iterative formulas of problems (3) and (4) are inferred as follows:

(5) $$m_{k}^{n+1\;}(w)=\frac{f(w)-\sum_{i\neq k}m_{i}(w)+\frac{\beta (w)}{2}}{1+2\alpha {\left(w-w_{k}\right)}^{2}}$$

(6) $$w_{k}^{n+1\;}=\frac{\int_{0}^{\infty }w{\left|m_{k}(w)\right|}^{2}dw}{\int_{0}^{\infty }{\left|m_{k}(w)\right|}^{2}dw}$$

The Lagrangian multiplier is renewed according to Equation (7):

(7) $$\beta^{n+1}=\beta^{n}+\tau (f-\sum_{i}m_{i})$$

where *τ* is the update parameter. The prime procedures of VMD can be summarized as follows:

*Step* 1: Initialize $m_{k}^{1}$, $w_{k}^{1}$, $\beta^{1}$ *n* = 1;
*Step* 2: Update *m_k_* and *w_k_* based on Equations (5) and (6);
*Step* 3: Update *β* based on Equation (7), *n* = *n* + 1;
*Step* 4: If $\sum_{k}{\left\|m_{k}^{n+1}-m_{k}^{n}\right\|}_{2}^{2}>\varepsilon$, turn to *Step 2*, else stop iterating.

### 2.2. Permutation Entropy

The principle of permutation entropy (PE) does not consider the specific values of the data. Instead, it is based on the comparison and reconstruction of adjacent data, which is simple in computation and has the advantage of anti-interference [44]. Given a time series $X=[x_{1},x_{2},\ldots ,x_{N}]$, the phase space is reconstructed firstly within PE algorithm as follows:

(8) $$\begin{array}{l} X_{1}=[x_{1},x_{1+\tau \;},\ldots ,x_{1+(m-1)\tau }]\\ \vdots \\ X_{i}=[x_{i},x_{i+\tau },\ldots ,x_{i+(m-1)\tau }]\\ \vdots \\ X_{N-(m-1)\tau }=[x_{N-(m-1)\tau },x_{N-(m-2)\tau },\ldots ,x_{N}] \end{array}$$

Gathering the above formulas, an overall matrix $X_{i}{}^{m}$ can be obtained:

(9) $$X_{i}{\;}^{m}={\left[X_{1},X_{2},\ldots ,X_{i},\ldots ,X_{N-(m-1)\tau }\right]}^{T}$$

where *m* is the embedded dimension with the integer scope [3, 7], while *τ* is time delay and generally set as the integer 1.

Each element in $X_{i}$ can be sorted in ascending order: $X_{i}=[x_{i+(j_{1}-1)\tau },\leq x_{i+(j_{2}-1)\tau },\leq \ldots ,\leq x_{i+(j_{m}-1)\tau }],$ where $j_{1},j_{2},\ldots ,j_{m}$ are the column indexes of each element before ordering. If two elements are equal, i.e., $X_{i+(j_{p}-1)\tau }=X_{i+(j_{q}-1)\tau }$, they will be sorted sequentially which means:

(10) $$\;if:\;j_{p}<j_{q}\;\;\;\;X_{i+(j_{p}-1)\tau \;}\leq X_{i+(j_{q}-1)\tau }$$

Therefore, a symbol sequence can be obtained for any $X_{i}$:

(11) $$S(l)=[j_{1},j_{2},\ldots ,j_{m}],\;1\leq l\leq m!\;$$

The *m*-dimensional phase space maps a total of m! different symbol sequences $\left[j_{1},j_{2},\ldots ,j_{m}\right]$, among which symbol sequence S(l) is an individual. Accordingly, we can calculate the probability $p_{l}$ of each symbol sequence, where $p_{l}$ is subject to $\sum_{l=1}^{m!}p_{l}=1$.

(12) $$p_{l}=\frac{n}{N-(m-1)\tau \;}$$

where *n* is the occurrence times of each symbol sequence S(l).

According to the above formula, PE of the time series *X* can be defined as follows in the form of information entropy:

(13) $$H_{P}(m)=-\sum_{l=1\;}^{m!}p_{l}\ln p_{l}$$

When $p_{l}=\frac{1}{m!}$, $H_{P}(m)$ reaches the maximum value ln(m!). For convenience, $H_{P}(m)$ is usually normalized by ln(m!):

(14) $$H_{P}=\frac{H_{P}(m)\;}{\ln(m!)}$$

where $H_{P}$ is in the range of [0, 1], which represents the randomness of the time series $X_{i}$, i.e., the larger $H_{P}$ is, the more random the time series is.

## 3. Pattern Recognition Based on SVM Optimized by CQSCA

### 3.1. Support Vector Machine

SVM is a data mining method based on structural risk minimization and statistical learning theory [35], whose core innovative idea is to map the sample space to a high-dimensional feature space through nonlinear kernel transformation. Owing to the optimal hyper-plane in the high-dimensional feature space, the nonlinear classification in sample space is realized by solving the linear classification of feature space, which makes SVM can successfully deal with nonlinear pattern recognition problems. Compared with traditional learning machines, SVM has an outstanding adaptability to limited samples and is not sensitive to data dimension.

Given a data set {(*x_i_*, *y_i_*), *i* = 1, 2, …, *n*} from two classes, there must exist a classification hyper-plane, the construction of which is the most important task for achieving pattern recognition with SVM. The hyper-plane can be formulated as:

(15) w⋅x+b=0 

where *w* and *b* represent the weight vector and bias term, respectively, while w⋅x is the inner product of *w* and *x*.

For a binary classification issue with labels −1 and 1, all the samples should meet a specific condition as defined in Equation (16), thus the two types of samples can be completely separated:

(16) $$w\cdot x_{i}+b\left\{ \begin{array}{l} >1\;\;for\;\;y_{i}=1\\ <-1\;\;for\;\;y_{i}=-1 \end{array}\;\right.$$

To solve linearly non-separable problems, slack variable *ξ* and penalty factor *C* are introduced, thus the generalized hyper-plane can be deduced by solving the following optimization problem:

(17) $$\left\{ \begin{array}{l} \min f=\frac{1}{2}{\|w\|\;}^{2}+C\sum_{i=1}^{n}\xi_{i}\\ s.t.\;\;\;y_{i}({\vec{w}}^{T}\cdot x_{i}+b)\geq 1-\xi_{i},\;\;\;\;\;\;\;i=1,2,\ldots ,n \end{array}\right.$$

During the process of mapping the samples into higher dimension space, radial basis kernel function is always employed which is defined as:

(18) $$K(x_{i},x_{j})=\phi (x_{i})\cdot \phi (x_{j})=\exp(-g||x_{i}-x_{j}|{|}^{2})\;$$

where *g* is the kernel parameter.

In accordance with Lagrange theory and duality principle, the dual form of optimization problem (17) can be reformulated as:

(19) $$\begin{array}{l} \max L=\sum_{i=1\;}^{n}\mu_{i}-\frac{1}{2}\sum_{i,j=1}^{n}\mu_{i}\mu_{j}y_{i}y_{j}K(x_{i},x_{j})\\ s.t.\;\;\;\sum_{i=1}^{n}\mu_{i}y_{i}=0,\;\;\mu_{i}\geq 0,\;\;\;\;\;\;i=1,2,\ldots ,n \end{array}$$

where *μ_i_* are Lagrange multipliers.

With the Lagrange multipliers acquired from the solution of the above dual problem, the decision function of the original problem can be ascertained:

(20) $$f(x)=sgn(\sum_{i=1\;}^{n}\mu_{i}K(x_{i},x)+b)$$

### 3.2. Chaos Quantum Sine Cosine Algorithm (CQSCA)

#### 3.2.1. Sine Cosine Algorithm

The optimization procedure of SCA includes two phases [40]: exploration and exploitation. During the exploration phase, the algorithm is firstly initialized with a collection of random solutions to start the optimization process. With the stochastic searching, SCA can locate feasible solutions quickly in the searching space. Meanwhile, in the exploitation phase, the random solutions change gradually and the changing rate is obviously lower than that during the exploration phase, which contributes to a better searching in current space.

The positions of *m* individuals are randomly generated in initialization phase of SCA. Supposing that each solution of the optimization problem corresponds to individual’s position in the searching space, and the position of i-th (i=1,2,…,m) individual is represented by $X_{i}={\left(X_{i1},X_{i2},\ldots ,X_{iD}\right)}^{T}$, where D is individual’s dimension. The individual *i*’s best value is $P_{i}={\left(P_{i1},P_{i2},\ldots ,P_{iD}\right)}^{T}$. The position of individual *i* will be updated by the following formulas in the iteration [40]:

(21) $$\begin{array}{l} X_{i}{\;}^{k+1}=X_{i}{}^{k}+r_{1}\times \sin(r_{2})\times |r_{3}P_{i}{}^{k}-X_{i}{}^{k}|\\ X_{i}{}^{k+1}=X_{i}{}^{k}+r_{1}\times \cos(r_{2})\times |r_{3}P_{i}{}^{k}-X_{i}{}^{k}| \end{array}$$

where $X_{i}{}^{k}$ is the position of individual *i* in the *k-*th iteration. The above equations can be combined as follows:

(22) $$X_{i}{\;}^{k+1}=\left\{ \begin{array}{l} X_{i}{}^{k}+r_{1}\times \sin(r_{2})\times |r_{3}P_{i}{}^{k}-X_{i}{}^{k}|,r_{4}<0.5\\ X_{i}{}^{k}+r_{1}\times \cos(r_{2})\times |r_{3}P_{i}{}^{k}-X_{i}{}^{k}|,r_{4}\geq 0.5 \end{array}\right.$$

As is shown in the above equations, four parameters are mainly included in the updating Equations [40]: $r_{1},r_{2},r_{3}$ and $r_{4}$. The parameter $r_{1}$ is a random number, dictating the next iteration position’s movement direction of individual *i*. The parameter $r_{2}$ is a random number in [0, 2π], which defines the distance that the movement should be towards or outwards the destination. To randomly emphasize ($r_{3}$ > 1) or deemphasize ($r_{3}$ < 1) the effect from the best value of individual during the movement, the parameter $r_{3}$ is brought with a random weight with the range of [0, 2]. Lastly, the parameter $r_{4}$ is a random number in [0, 1] to switch equally between components, when $r_{4}$ < 0.5, the position of individual *i* iterates by sine component, otherwise iteration switches to cosine component.

During the searching process, SCA should balance the exploration and exploitation phases and finally find the global optimum in the searching space. Accordingly, the amplitudes of the sine and cosine functions are adaptively changed by adjusting $r_{1}$ in the updating Equation [40]:

(23) $$r_{1}=a-t\frac{a}{T}\;$$

where *T*, *t* and *a* are respectively the maximum number of iterations, the current number of iterations and constant.

#### 3.2.2. Quantum Sine Cosine Algorithm

QSCA is the improved version of SCA with quantum evolution [45]. In quantum description, the smallest unit of information is a qubit, and any state of a qubit can be represented as a linear combination of the basic states, called superposition |ϕ〉. The qubit can also be expressed by probability amplitude $|\phi \rangle ={\left[\cos(\theta ),\sin(\theta )\right]}^{T}$, where *θ* is the phase of a qubit. The probability amplitude of the qubit is directly used as the encoding of the solution vector to avoid the randomness of the transformation in QSCA [45]. The coding pattern is:

(24) $$p_{i}=\left[\begin{array}{l} \cos(\theta_{i1\;})\\ \sin(\theta_{i1}) \end{array}|\begin{array}{l} \cos(\theta_{i2})\\ \sin(\theta_{i2}) \end{array}|\ldots |\begin{array}{l} \cos(\theta_{iD})\\ \sin(\theta_{iD}) \end{array}\right]$$

where $\theta_{ij}=2\pi \;\times \;rand$, *rand* is a random number in [0, 1], i=1,2,…,m, *m* is the size of populations, j=1,2,…,D, *D* is the spatial dimension. As is shown in formula (24), each individual occupies two positions in the space:

(25) $$\begin{array}{l} p_{i}{\;}^{c}=[\cos(\theta_{i1}),\cos(\theta_{i2}),\ldots ,\cos(\theta_{iD})]\\ p_{i}{}^{s}=[\sin(\theta_{i1}),\sin(\theta_{i2}),\ldots ,\sin(\theta_{iD})] \end{array}$$

For convenient expression, $p_{i}{}^{c}$ is called cosine position, while $p_{i}{}^{s}$ is called sine position. Since the individual’s traversal scope is [−1, 1] in every dimension, the two positions occupied by the individuals need to be mapped to the solution space of the corresponding optimization problem. Each probability amplitude of an individual qubit corresponds to an optimization variable in the solution space. As the *j-*th qubit of the individual *i* is ${\left[\cos(\theta ),\sin(\theta )\right]}^{T}$, the corresponding solution space variable is in [45]:

(26) $$\begin{array}{l} X_{ij\;}{}^{c}=\frac{1}{2}[b_{j}(1+\cos(\theta_{ij}))+a_{j}(1-\cos(\theta_{ij}))]\\ X_{ij}{}^{s}=\frac{1}{2}[b_{j}(1+\sin(\theta_{ij}))+a_{j}(1-\sin(\theta_{ij}))] \end{array}$$

During the status updating stage for all individuals, the movement of an individual’s position is implemented by a quantum rotation gate. The individual’s position will move according to the following rules:

(1) The qubit updating of phase increment for individual *i*:

(27) $$\Delta \theta_{ij\;}{}^{k+1}=\left\{ \begin{array}{l} r_{1}\times \sin(r_{2})\times \Delta \theta_{g},r_{4}<0.5\\ r_{1}\times \cos(r_{2})\times \Delta \theta_{g},r_{4}\geq 0.5 \end{array}\right.$$

where $\Delta \theta_{g}=\left\{ \begin{array}{l} 2\pi +\theta_{gj}-\theta_{ij},\;\theta_{gj}-\theta_{ij}<-\pi \\ \theta_{gj}-\theta_{ij},\;\;\;-\pi \leq \theta_{gj}-\theta_{ij}\leq \pi \\ \theta_{gj}-\theta_{ij}-2\pi ,\;\theta_{gj}-\theta_{ij}>\pi \end{array}\right.$.

(2) The qubit updating of probability amplitude for individual *i*:

(28) $$\left[\begin{array}{l} \cos(\theta_{ij\;}{}^{k+1})\\ \sin(\theta_{ij}{}^{k+1}) \end{array}\right]=\left[\begin{array}{ll} \cos(\Delta \theta_{ij}{}^{k+1}) & -\sin(\Delta \theta_{ij}{}^{k+1})\\ \sin(\Delta \theta_{ij}{}^{k+1}) & \cos(\Delta \theta_{ij}{}^{k+1}) \end{array}\right]\left[\begin{array}{l} \cos(\theta_{ij}{}^{k})\\ \sin(\theta_{ij}{}^{k}) \end{array}\right]=\left[\begin{array}{l} \cos(\theta_{ij}{}^{k}+\Delta \theta_{ij}{}^{k+1})\\ \sin(\theta_{ij}{}^{k}+\Delta \theta_{ij}{}^{k+1}) \end{array}\right]$$

After the above two updating processes, the two new positions are formulated as:

(29) $$\begin{array}{l} P_{i}{{}^{\prime }}^{c}=(\cos(\theta_{i1}{}^{k}+\Delta \theta_{i1}{}^{k+1}),\cos(\theta_{i1}{}^{k}+\Delta \theta_{i2}{}^{k+1}),\ldots ,\cos(\theta_{iD}{}^{k}+\Delta \theta_{iD}{}^{k+1}))\\ P_{i}{{}^{\prime }}^{s}=(\sin(\theta_{i1}{}^{k}+\Delta \theta_{i1}{}^{k+1}),\sin(\theta_{i1}{}^{k}+\Delta \theta_{i2}{}^{k+1}),\ldots ,\sin(\theta_{iD}{}^{k}+\Delta \theta_{iD}{}^{k+1})) \end{array}$$

To increase the diversity of population and avoid local optimum, a mutation operator with quantum non-gate is introduced in Reference [45]. Firstly, a random number within (0, 1) is created and compared with the given mutation probability $p_{m}$ for each individual. Then, a total number of 0.5*D* qubits from each individual are randomly selected, whose probability amplitudes are changed by quantum non-gate if $rand_{i}<p_{m}$, otherwise, the amplitude phase remains unchanged:

(30) $$[\begin{array}{ll} 0 & 1\\ 1 & 0 \end{array}]\;\left[\begin{array}{l} \cos(\theta_{ij})\\ \sin(\theta_{ij}) \end{array}\right]=\left[\begin{array}{l} \sin(\theta_{ij})\\ \cos(\theta_{ij}) \end{array}\right]=\left[\begin{array}{l} \cos(\frac{\pi }{2}-\theta_{ij})\\ \sin(\frac{\pi }{2}-\theta_{ij}) \end{array}\right]$$

The procedures of QSCA are detailed as follows [45]:

*Step 1*: Initialize the population and set relevant parameters according to Equation (24);
*Step 2*: Transform unit space to solution space on the basis of Equation (26), thus to calculate the fitness of each individual;
*Step 3*: Update individual’s status with Equations (27) and (28);
*Step 4*: Implement the mutation process based on the given mutation probability according to Equation (30);
*Step 5*: Loop steps 2–4 until the convergence condition is met or the maximum times of iterations is reached.

#### 3.2.3. Chaos Quantum Sine Cosine Algorithm

Chaos is a kind of seemingly irregular and random phenomenon happening within nonlinear systems resulted from deterministic rules. It appears to be disorganized but has certain motion laws, representing the complexity, randomness, and disorder within the systems. Chaotic variables have the features of pseudo-randomness and ergodicity, which traverses all points in a certain scope of the solution space without repeatability. The basic idea of searching with chaotic variables is to make full use of the ergodicity, which means that some chaotic variables are created with a chaotic map and transformed to the range of variables to be optimized, then the optimal parameters are searched [46]. With the chaotic variables, it would be more likely to find the global optimum. To promote the searching performance of QSCA, a Duffing system [47] is employed to create the chaotic variables. The dynamical equation of Duffing system is given by:

(31) $$x^{\prime \prime }(t)+\gamma x^{\prime }(t)-\alpha x(t)+\beta x^{3}(t)=A\cos(wt)\;$$

where coefficient *γ* is the damping degree, *α* is the toughness degree, *β* is the nonlinearity of power, *A* is the amplitude of driving force, *ω* is the circular frequency of driving force. The differential form of Equation (31) can be obtained by transformation, which are given by:

(32) $$\begin{array}{l} x^{\prime }(t)=y(t)\\ y^{\prime }(t)=-\gamma y(t)+\alpha x(t)-\beta x^{3}(t)+A\cos(wt) \end{array}$$

The coefficients of the Duffing system except driving force *A* are chosen as *γ* = 0.1, *α* = 1, *β* = 0.25, *ω* = 2. Given the initial values x(0) and y(0), the system’s status will evolve gradually with the value of driving force *A* changing. When the dynamic behavior of the Duffing system is chaotic, chaotic variables *x* and *y* will traverse the points in a certain scope. Then, some points from the traversed ones are selected at a certain interval, after which a linear transformation from the chaotic variables space to the solution space is executed, thus to produce the initial solutions $X_{i}$, i=1,2,…,m of QSCA. 

### 3.3. SVM Optimized by CQSCA

The main procedures of the optimized SVM with the proposed chaos quantum sine cosine algorithm (CQSCA) are as follows:

*Step 1*: Create chaotic variables by a Duffing system based on Equation (31) and transform them to the range of [0, 1];
*Step 2*: Initialize the population with the processed chaotic variables;
*Step 3*: Encode the quantum and transform unit space to solution space;
*Step 4*: Calculate the fitness of each individual, i.e., the cross-validation accuracy of SVM;
*Step 5*: Update individual’s status with Equations (27) and (28);
*Step 6*: Implement mutation process based on the given probability according to formula (31);
*Step 7*: Loop steps 3–6 until the convergence condition is met or the maximum number of iterations is reached;
*Step 8*: Choose *C* and *g* in accordance with the maximal cross-validation accuracy as the optimal parameters;
*Step 9*: Train the optimal SVM model with the training set;
*Step 10*: Recognize the testing set.

**Figure 1:**
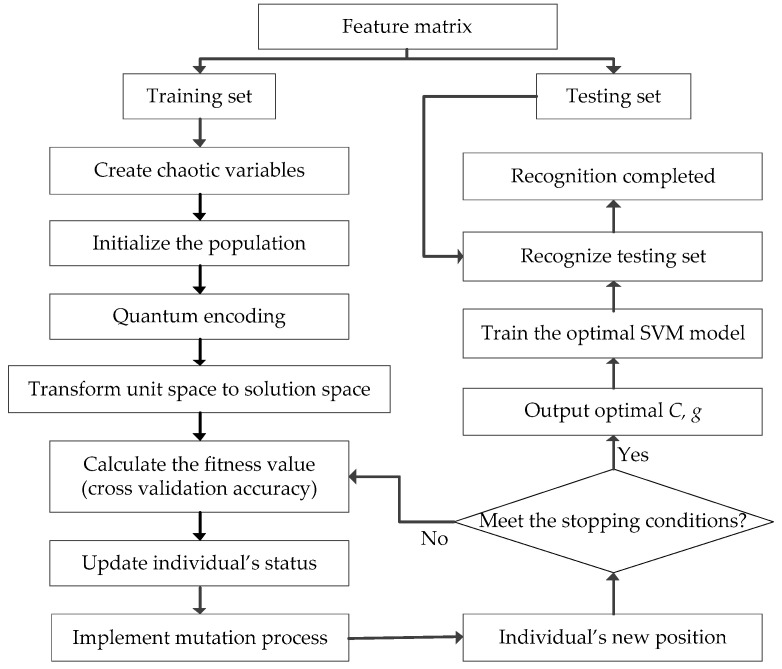
The flowchart of SVM optimized by CQSCA.

The flowchart of SVM optimized by CQSCA is shown in Figure 1.

### 3.4. Pattern Recognition Experiments

To estimate the performance of the proposed method, some standard UCI datasets [48] including wine, iris and heart were selected for pattern recognition experiment. The basic information of the datasets is shown in Table 1. All attributes of the datasets were normalized to be in the range [0, 1]. 

Five-fold cross-validation was utilized to search the optimal parameters of *C* and *g*, which means that all the three datasets were haphazardly divided into five subsets, and each time one subset was selected as testing data while the other four ones as training data. The searching scopes of *C* and *g* were both [2^−10^, 2^10^]. The numbers of individuals and iterations were set as 30 and 100, respectively. The constant *a* for changing the amplitudes of the sine and cosine functions was set as 2. The mutation probability $p_{m}$ was set as 0.04. 

In order to compare with the proposed method (SVM-CQSCA), SVM optimized by PSO (PSO-SVM) and SVM optimized by SCA (SCA-SVM) were employed. The searching scopes of parameters *C* and *g* for SCA-SVM and PSO-SVM were the same as the configuration for SVM-CQSCA. The constant *a* in SCA-SVM was set as 2. To measure the performance of all methods well, the experiment was rerun for totally ten times. In each experiment, the best *C* and *g* were determined based on the maximal cross-validation accuracy, then the SVM model was trained and applied to classify all the data. 

**Figure 2:**
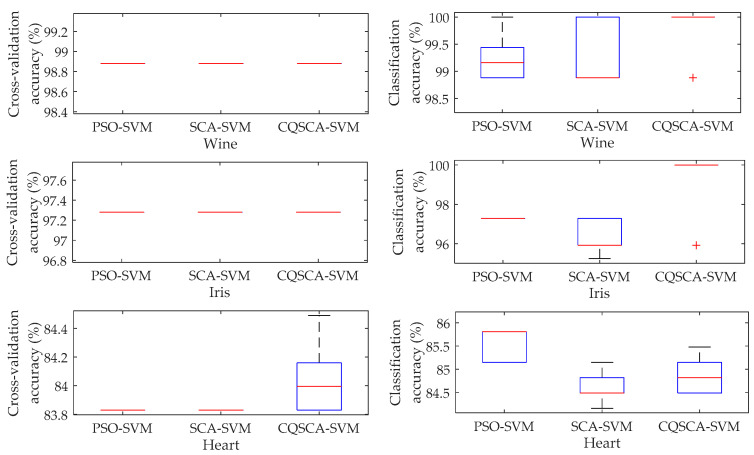
Boxplots of recognition results with different recognition methods.

The experimental results are shown in Table 2, where the cross-validation accuracy and classification accuracy both donate the average of all results. The parameters *C* and *g* are corresponding to the best cross-validation accuracy. Meanwhile, the deviation scope is employed for error analysis in accordance with the mean value. Additionally, boxplots are employed to reveal the performance of different methods visually in Figure 2. As the results show, the proposed method achieves better classification performance than other methods by introducing a Duffing system for chaotic initialization and quantum technique for improving the optimization efficiency. 

## 4. Hybrid Fault Diagnosis Based on VMD and SVM Optimized by CQSCA

The procedures of the proposed hybrid fault diagnosis approach with entropy-based feature extraction and SVM optimized by chaos quantum sine cosine algorithm (CQSCA) are detailed as follows:

*Step 1*: Collect the vibration signals;
*Step 2*: Select the mode number *K* of VMD through center frequency observation method;
*Step 3*: Decompose all fault samples into sets of IMFs with VMD;
*Step 4*: Calculate the PEs of all IMFs;
*Step 5*: Construct the fault feature vectors with the PEs for all fault samples;
*Step 6*: Search the optimal parameters *C* and *g* for SVM with the proposed CQSCA;
*Step 7*: Train SVM with the optimal parameters *C* and *g*, thus the optimized SVM model is obtained;
*Step 8*: Apply the optimal SVM model to recognize different types of faults.

**Figure 3:**
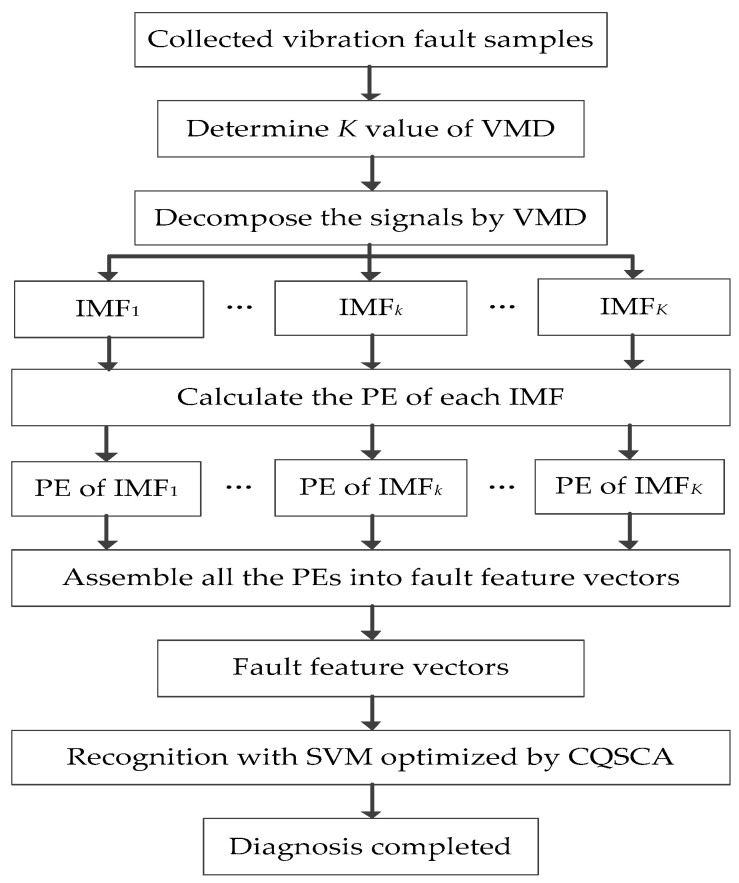
Flowchart of the proposed hybrid fault diagnosis approach.

The flowchart of the proposed hybrid fault diagnosis approach is shown in Figure 3.

## 5. Engineering Application

### 5.1. Data Collection

**Figure 4:**
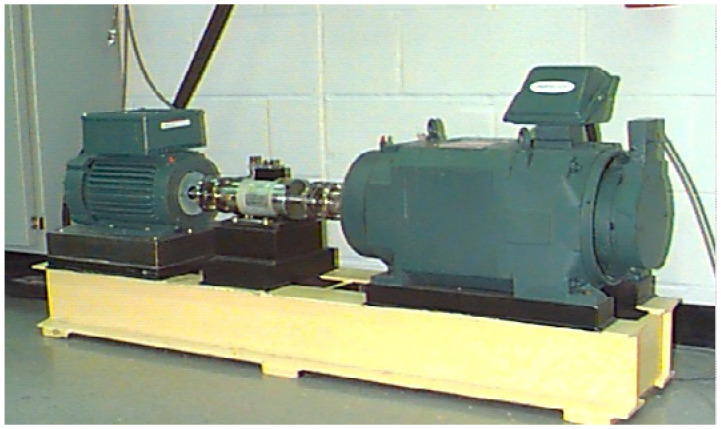
Experiment device in bearing data center.

The experimental data gathered from Bearings Data Center of Case Western Reserve University [49] were employed to validate the capability of the proposed method in this paper. As shown in Figure 4, the experiment device mainly consists of a motor, an accelerometer and a torque sensor/encoder. The bearing is a SKF deep groove ball bearing model 6205-2RS. Accelerometers were placed at the end of the motor housing for data acquisition. The bearing data was collected from the drive end (DE). The inner, outer and ball element diameters of the bearing were 0.9843, 2.0472 and 0.3126 inches respectively, and the number of ball elements is nine. Single point faults were introduced to the test bearings by using electro-discharge machining, simulating the four working states of the rolling bearing: normal state, inner race fault, outer race fault and ball element fault. The fault diameters were 0.007 inches and 0.021 inches with the depth of 0.011 inches. In the experiment, the rotation speed was 1797 rpm under the load of 0 hp and the sample frequency was 12,000 Hz. The samples used in this paper include 7 types, namely normal state, outer race fault, inner race fault and ball fault with diameters of 0.007 inches and 0.021 inches (i.e., each of the three types of faults has two defect sizes). In addition, all data were partitioned into 59 segments containing 1024 sampling points for each type of signals. Details of the experimental data are listed in Table 3. 

### 5.2. Engineering Application

To verify the effectiveness of the proposed VMD-PE-CQSCA-SVM method, the experiment was conducted with the comparison of EMD and EEMD during the signal decomposing phase. Likewise, when optimizing the parameters *C* and *g* for SVM, PSO and SCA are employed for comparison. In other words, eight different methods were applied to achieve the contrastive analysis, including EMD-PE-PSO-SVM, EMD-PE-SCA-SVM, EMD-PE-CQSCA-SVM, EEMD-PE-PSO-SVM, EEMD-PE-SCA-SVM, EEMD-PE-CQSCA-SVM, VMD-PE-PSO-SVM, VMD-PE-SCA-SVM. When decomposing the fault samples with VMD, the decomposing mode number *K* needs to be preset. If the value of *K* is too small, the reduction of non-stationarity for original signal is limited. On the contrary, when the value of *K* is too large, the center frequencies of adjacent components will be close to each other, resulting in mode mixing. In our application, a detected signal under inner race fault with diameter of 0.007 inches was applied to ascertain the parameter *K*. The normalized center frequencies of all IMFs with different *K* are listed in Table 4. As it can be seen from Table 4, similar normalized center frequencies appeared when *K* was set 5, i.e., excessive decomposition occurred. Hence, the total number of modes was set 4. 

**Figure 5:**
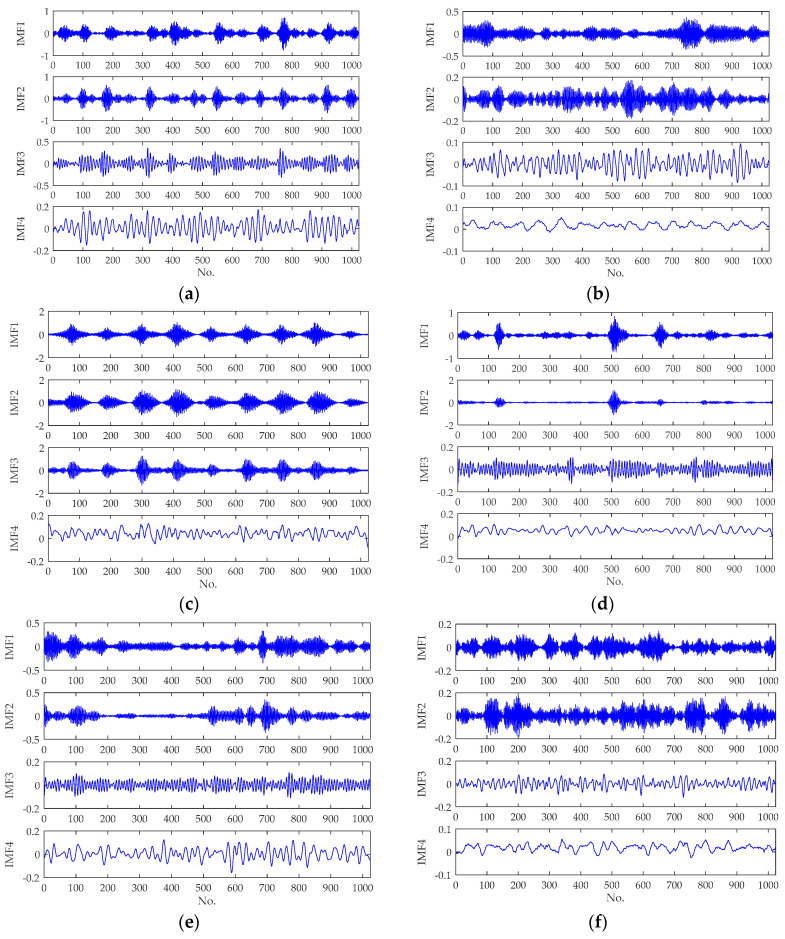

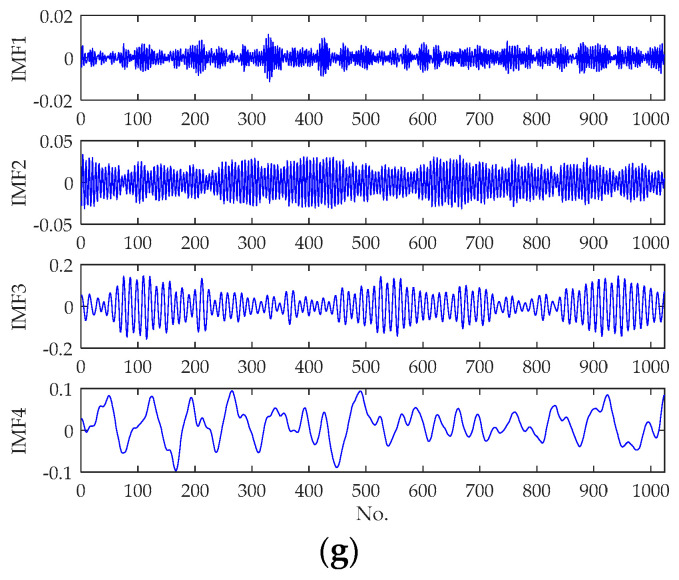
The VMD decomposition results of signals from different working states: (**a**) fault-inner race (0.007 inches); (**b**) fault-ball (0.007 inches); (**c**) fault-outer race (0.007 inches); (**d**) fault-inner race (0.021 inches); (**e**) fault-ball (0.021 inches); (**f**) fault-outer race (0.021 inches); (**g**) normal state.

The decomposition results of signals from different kind of working states are shown in Figure 5, from which it can be seen that the original non-stationary signals were decomposed into four components with different frequency bands by VMD. As the time domain waveforms shown in Figure 5, it is of apparent difference among the decomposing results from different kind of working states. After decomposing the vibration signals, the PEs were calculated for each component to constitute the fault feature vector. During the calculation of PEs, the embedded dimension *m* and the time delay *τ* were set as 3 and 1 respectively. The PEs of five samples from different type of signals (L0–L6) are listed in Table 5.

Among the 59 feature vectors from each kind of operational condition, 40 were randomly selected as training samples, while the other 19 ones were selected for testing. The penalty parameter *C* and the kernel parameter *g* of SVM were optimized by the proposed CQSCA which had 30 particles and iterated 100 times, where the searching ranges of *C* and *g* were both $\left[2^{-10},2^{10}\right]$. During the optimization process, the fitness value was measured with the five-fold cross validation accuracy. Then, the SVM model with the selected optimal parameters *C* and *g* was trained and employed to recognize the testing samples. In order to further verify the availability of the proposed method, the experiment was repeated ten times and the training samples were selected randomly every time, after which the average accuracy and corresponding deviation scope were calculated to evaluate the performance in both training and testing phases. Furthermore, the optimal (*C*, *g*) is reported corresponding to the best training accuracy.

In comparative experiment, all components decomposed by EMD and EEMD were employed to calculate the PEs, during which process the parameter setting for PE calculation was the same as the proposed method. The optimum parameters *C* and *g* in all the eight contrastive methods are decided the alike way as done in proposed method, i.e., 30 particles and 100 iterations are presented, while the search scopes of *C* and *g* are both $\left[2^{-10},2^{10}\right]$. In addition, the way of performance evaluation was the same as done in the proposed method as well.

**Figure 6:**
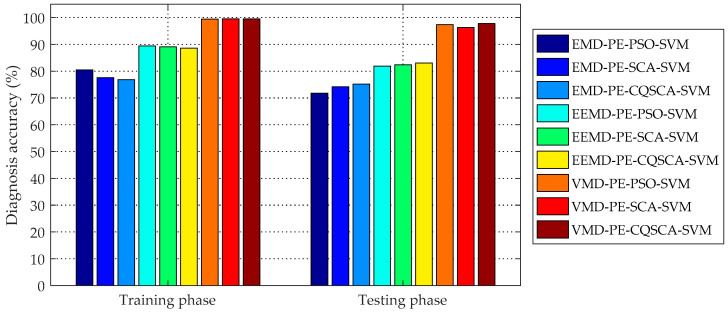
Comparison of all experimental results with different methods.

**Figure 7:**
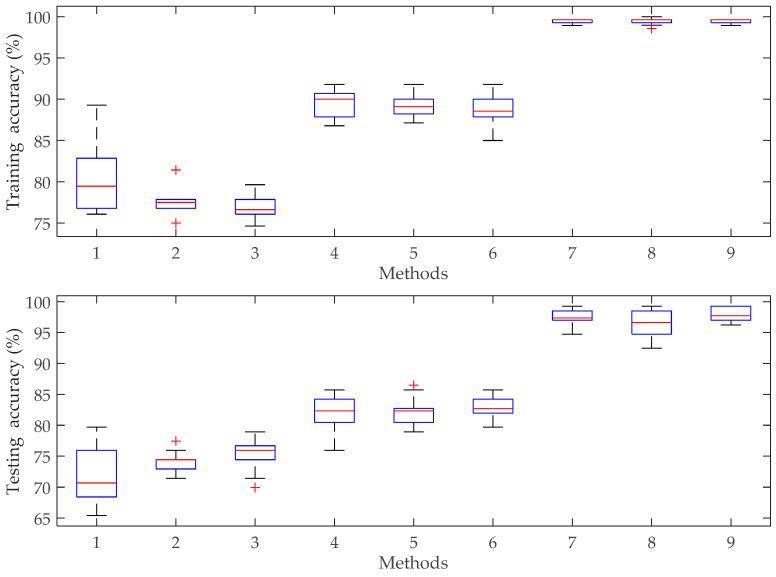
Boxplots of diagnosis results with different methods, the x-axis tick labels correspond to: 1: EMD-PE-PSO-SVM; 2: EMD-PE-SCA-SVM; 3: EMD-PE-CQSCA-SVM; 4: EEMD-PE-PSO-SVM; 5: EEMD-PE-SCA-SVM; 6 EEMD-PE-CQSCA-SVM; 7: VMD-PE-PSO-SVM; 8: VMD-PE-SCA-SVM; 9: VMD-PE-CQSCA-SVM.

The fault diagnosis results and comparison of accuracies with different methods are presented in Table 6 and Figure 6. From Table 6, it can be viewed that the proposed VMD-PE-CQSCA-SVM method achieved the best precision in both training and testing phases, i.e., 99.50% and 97.89%, respectively. Specifically, it can be seen from the comparison of EMD-PE-CQSCA-SVM, EEMD-PE-CQSCA-SVM and VMD-PE-CQSCA-SVM that the testing accuracy of the proposed method is respective 22.70% and 14.88% higher than that of the other two methods, which shows the fact that VMD, as a non-stationary signal processing method, can promote the fault representation ability of PE. Furthermore, the contrastive analysis among VMD-PE-PSO-SVM, VMD-PE-SCA-SVM and VMD-PE-CQSCA-SVM shows that CQSCA-optimized SVM improved the accuracy by 0.52% and 1.57% than PSO-optimized SVM and SCA-optimized SVM respectively, indicating the availability of the proposed CQSCA optimizing strategy. Additionally, boxplots are employed to reveal the performance of different diagnosis methods visually in Figure 7, from which it can be seen that the proposed VMD-PE-CQSCA-SVM method achieves better precision and stability than other contrastive methods.

## 6. Conclusions

In order to enhance the fault diagnosis precision for rotating machinery, a hybrid approach with the fusion of entropy-based feature extraction and SVM optimized by a chaos quantum sine cosine algorithm is proposed in this paper. Firstly, the preset parameter *K* of VMD is chosen using the central frequency observation method, after which the signals collected under different states are decomposed into series of intrinsic mode functions (IMFs). Subsequently, the permutation entropy values of all IMFs are calculated to assemble the feature vectors of different fault samples. Finally, an optimized SVM model based on chaotic initialization, quantum technique and SCA (CQSCA) for parameter selection, whose availability has been ascertained with recognizing experiment, is proposed to achieve the pattern recognition for different kind of faults. In the engineering applications, the proposed VMD-PE-CQSCA-SVM method was successfully employed to recognize different fault samples and compared with some other relevant methods, including EMD-PE-PSO-SVM, EMD-PE-SCA-SVM, EMD-PE-CQSCA-SVM, EEMD-PE-PSO-SVM, EEMD-PE-SCA-SVM, EEMD-PE-CQSCA-SVM, VMD-PE-PSO-SVM, VMD-PE-SCA-SVM. The application results indicate that the proposed method achieves the best performance during both the training stage and testing stage in terms of the average accuracy of ten times randomized experiments. Particularly, the test accuracy of the proposed method is 22.70% and 14.88% higher than that of EMD-PE-CQSCA-SVM and EEMD-PE-CQSCA-SVM, and also 0.52% and 1.57% higher than VMD-PE-PSO-SVM and VMD-PE-SCA-SVM. Furthermore, the boxplots of different diagnosis methods show that the stability and precision of the proposed method is superior to those of other methods. Thus, the proposed method is a reliable and effective tool for fault diagnosis of rotating machinery.

## Figures and Tables

**Table 1:** The basic information of the datasets.

Dataset	Number of Attributes	Number of Classes	Number of Data
Wine	13	3	178
Iris	4	3	150
Heart	13	2	303

**Table 2:** Pattern recognition results with different methods.

Methods	Dataset	*C*	*g*	Accuracy (%)	
				Cross-Validation	Classification
PSO-SVM	Wine	0.6629	2.9830	98.88, [0, 0]	99.27, [−0.39, 0.75]
	Iris	426.2439	0.0010	97.28, [0, 0]	97.28, [0, 0]
	Heart	66.8865	0.0010	83.83, [0, 0]	85.54, [−0.40, 0.22]
SCA-SVM	Wine	603.7267	4.9808	98.88, [0, 0]	99.21, [−0.34, 0.75]
	Iris	490.5731	0.0010	97.28, [0, 0]	96.33, [−1.09, 0.84]
	Heart	78.8959	0.0013	83.83, [0, 0]	84.65, [−0.50, 0.48]
CQSCA-SVM	Wine	508.2523	5.3278	98.88, [0, 0]	99.89, [−1.01, 0.12]
	Iris	600.7378	31.3525	97.28, [0, 0]	99.59, [−3.67, 0.45]
	Heart	0.6663	0.1195	84.06, [−0.23, 0.41]	84.82, [−0.33, 0.63]

**Table 3:** Description of the experimental data.

Position of Fault	Defect Size (Inches)	Label of Classes	Number of Samples
Normal	-	L0	59
Inner race	0.007	L1	59
Ball	0.007	L2	59
Outer race	0.007	L3	59
Inner race	0.021	L4	59
Ball	0.021	L5	59
Outer race	0.021	L6	59

**Table 4:** Normalized center frequencies with different *K* value.

Number of Modes	Normalized Center Frequencies						
2	0.2221	0.0860					
3	0.2981	0.2253	0.0952				
4	0.2982	0.2260	0.1121	0.0400			
5	0.3041	0.2772	0.2238	0.1140	0.0494		
6	0.3047	0.2813	0.2358	0.2100	0.1099	0.0490	
7	0.3152	0.2992	0.2780	0.2357	0.2102	0.1096	0.0490

**Table 5:** Permutation entropy of different fault samples.

Fault Label	Sample Number	Permutation Entropy for Different Imf			
		IMF1	IMF2	IMF3	IMF4
L0	1	0.9629	0.8838	0.7110	0.5392
	2	0.9713	0.8836	0.7111	0.5282
	3	0.9589	0.8833	0.7132	0.5395
	4	0.9468	0.8830	0.7146	0.5351
	5	0.8836	0.7146	0.6281	0.5304
L1	1	0.9931	0.9472	0.7797	0.6424
	2	0.9941	0.9465	0.7839	0.6668
	3	0.9947	0.9476	0.7791	0.6485
	4	0.9947	0.9425	0.7843	0.6350
	5	0.9941	0.9486	0.7835	0.6539
L2	1	0.9853	0.9592	0.6593	0.7678
	2	0.9850	0.9518	0.6720	0.7502
	3	0.9871	0.9576	0.6678	0.7311
	4	0.9854	0.9557	0.6921	0.7546
	5	0.9859	0.9510	0.6773	0.7315
L3	1	0.9925	0.9877	0.9598	0.7015
	2	0.9928	0.9877	0.9599	0.7092
	3	0.9930	0.9871	0.9598	0.7515
	4	0.9926	0.9869	0.9578	0.7079
	5	0.9930	0.9879	0.9595	0.7437
L4	1	0.9926	0.9501	0.7869	0.6663
	2	0.9913	0.9508	0.7945	0.6815
	3	0.9942	0.9521	0.6892	0.7902
	4	0.9934	0.9510	0.7955	0.6602
	5	0.9938	0.9501	0.7859	0.7211
L5	1	0.9811	0.9475	0.7869	0.6203
	2	0.9828	0.9465	0.7884	0.6141
	3	0.9801	0.9448	0.7990	0.6117
	4	0.9819	0.9507	0.7875	0.6456
	5	0.9818	0.9523	0.7887	0.6382
L6	1	0.9965	0.9866	0.7094	0.7709
	2	0.9980	0.9888	0.7340	0.7959
	3	0.9970	0.9877	0.7130	0.7883
	4	0.9985	0.9880	0.7927	0.6727
	5	0.9984	0.9881	0.7983	0.6564

**Table 6:** Fault diagnosis results with different methods.

Methods	*C*	*g*	Diagnosis Accuracy (%)	
			Training Phase	Testing Phase
EMD-PE-PSO-SVM	438.1992	2.5166	80.46, [−4.40, 8.81]	71.80, [−6.39, 7.78]
EMD-PE-SCA-SVM	864.9884	1.5184	77.57, [−2.57, 3.78]	74.21, [−2.78, 3.40]
EMD-PE-CQSCA-SVM	769.6280	0.2422	76.86, [−2.22, 2.74]	75.19, [−5.27, 3.76]
EEMD-PE-PSO-SVM	192.4239	0.5464	89.46, [−2.67, 2.45]	81.88, [−5.94, 2.71]
EEMD-PE-SCA-SVM	316.7508	0.1267	89.07, [−1.93, 1.20]	82.41, [−3.46, 3.87]
EEMD-PE-CQSCA-SVM	1023.7402	0.0912	88.57, [−3.57, 3.22]	83.01, [−3.31, 2.83]
VMD-PE-PSO-SVM	265.3060	5.5333	99.45, [−0.57, 0.16]	97.37, [−2.63, 1.84]
VMD-PE-SCA-SVM	1024.0000	7.5830	99.50, [−0.93, 0.48]	96.32, [−3.84, 3.15]
VMD-PE-CQSCA-SVM	90.9980	7.2720	99.50, [−0.57, 0.16]	97.89, [−1.65, 1.42]
